# Temperature-related mortality estimates after accounting for the cumulative effects of air pollution in an urban area

**DOI:** 10.1186/s12940-016-0164-6

**Published:** 2016-07-11

**Authors:** Svetlana Stanišić Stojić, Nemanja Stanišić, Andreja Stojić

**Affiliations:** Faculty of Physical Chemistry, University of Belgrade, Studentski Trg 12-16, 11000 Belgrade, Serbia; Singidunum University, Danijelova 32, 11010 Belgrade, Serbia; Institute of Physics Belgrade, University of Belgrade, Pregrevica 118, 11080 Belgrade, Serbia

**Keywords:** Air pollution, Temperature, Mortality, Environmental exposure, Developing countries

## Abstract

**Background:**

To propose a new method for including the cumulative mid-term effects of air pollution in the traditional Poisson regression model and compare the temperature-related mortality risk estimates, before and after including air pollution data.

**Results:**

The analysis comprised a total of 56,920 residents aged 65 years or older who died from circulatory and respiratory diseases in Belgrade, Serbia, and daily mean PM_10_, NO_2_, SO_2_ and soot concentrations obtained for the period 2009–2014. After accounting for the cumulative effects of air pollutants, the risk associated with cold temperatures was significantly lower and the overall temperature-attributable risk decreased from 8.80 to 3.00 %. Furthermore, the optimum range of temperature, within which no excess temperature-related mortality is expected to occur, was very broad, between −5 and 21 °C, which differs from the previous findings that most of the attributable deaths were associated with mild temperatures.

**Conclusions:**

These results suggest that, in polluted areas of developing countries, most of the mortality risk, previously attributed to cold temperatures, can be explained by the mid-term effects of air pollution. The results also showed that the estimated relative importance of PM_10_ was the smallest of four examined pollutant species, and thus, including PM_10_ data only is clearly not the most effective way to control for the effects of air pollution.

**Electronic supplementary material:**

The online version of this article (doi:10.1186/s12940-016-0164-6) contains supplementary material, which is available to authorized users.

## Background

Environmental stressors, such as extreme temperature events and air pollution, pose a significant challenge to human societies, particularly to the growing urban population worldwide [1]. While most studies investigating the adverse health effects of air pollution have adjusted for temperature as a confounder, accounting for the effects of air pollution in the studies aimed at assessing the relationship between temperature and mortality has been less common [2]. The relatively few studies considering the interactive effects of meteorological variables and air pollution on daily mortality have reported inconsistent results [3, 4]. Significant inconsistency in the effect estimates across studies is at least partly associated with the methodological differences in exposure assessment and confounder control [5]. Furthermore, the majority of studies assessed air pollution-related mortality by including lag-specific effects of pollutants, despite the fact that detrimental impact of air pollution is not limited to few days preceding adverse health outcome, particularly in highly polluted areas of developing countries.

Better understanding of the temperature-related mortality is urgently needed, when considering the upcoming atmospheric changes and projected global temperature rise of 1.8 to 4.0 °C by 2100 [6]. The relationships between temperature and mortality were reported to be linear or non-linear, with death rates increasing in either direction from an optimum value [7, 8]. However, the relative importance of cold spells and heat waves still remains an issue of scientific debate. The latest study of Gasparrini et al. [9] including more than 74 million deaths recorded in 13 countries and 384 locations showed that cold-related deaths outnumbered heat-related deaths by a factor of nearly 20, and that most of the temperature-related mortality burden was attributable to milder, non-optimum weather, and not to extreme temperature events. Nevertheless, it should be emphasized that factors such as susceptibility or resilience, including air pollutants, in the most of cases have not been included in the analysis [10].

The evidence on the effects of environmental stressors in developing countries is relatively scarce because of poor environmental monitoring and a paucity of health surveillance data [11]. In this study, we compared the relative contributions from heat and cold to circulatory and respiratory mortality burden, before and after accounting for the cumulative effects of air pollution, as estimated by the method proposed herein. The study is based on data from Belgrade, Serbia, which is among the most polluted European cities [12, 13] and has recorded very high cardiovascular death rates over the last 15 years, ranging from 635 to 677 per 100,000 inhabitants [14, 15].

## Methods

The mortality data were stratified into age categories, and only people aged 65 and older were included in the analysis in order to increase the statistical power. Daily data of circulatory and respiratory mortality (cause-of-death coding I00-I99 and J00-J99 according to the International Classification of Diseases 10th revision, ICD-10 code) for the period from 2009–2014 were obtained from the Institute of Public Health Belgrade. Temperature data was obtained from the Global Data Assimilation System with spatial resolution of 1° and combined over 24-h periods to provide mean values.

In order to examine the associations between environmental stressors and mortality, two separate models were specified. Namely, the first model included temperature and other factors affecting mortality, such as season and day of the week, while the second, along with the above-mentioned factors, included air pollutants: PM_10_, NO_2_, SO_2_ and soot. The daily mean pollutant concentrations of PM_10_, NO_2_, SO_2_ and soot were obtained from 7, 21, 22 and 16 monitoring stations, respectively, uniformly distributed throughout the city area, within the monitoring network of the Institute of Public Health Belgrade. Measurements were conducted at automatic stations using a beta-ray attenuation sampler (Thermo FH 62 I-R) for PM_10_ and referent sampling devices, Horiba APNA 360 and APSA 360 analysers, for NO_2_ and SO_2_, respectively. At semi-automatic measuring stations, PM_10_ concentrations were determined using a referent Sven Leckel sampler, whereas determination of NO_2_ and SO_2_ concentrations was performed based on ISO 6768:1998 (Modified Griess-Saltzman method) and ISO 6767:1990 (tetrachloromercurate (TCM)/pararosaniline method) standards, respectively. Soot concentrations were obtained by the use of a Pro-Ekos device based on ISO 9835:1993 standard.

The effect of temperature on mortality is shown to be both delayed in time and shifted by a so-called harvesting effect or mortality displacement – a temporal advance in deaths occurring in the frailest population, typically followed by a period of reduced mortality [6]. In order to simultaneously capture the non-linear exposure-response dependencies and determine the time period between the exposure and health outcome, we used distributed lag non-linear models (DLNM). This methodology is based on the concept of cross-basis, a bidimensional space of functions that captures simultaneously the shape of the relationship between a predictor and a dependent variable along the space of the predictor and the lag dimension of its occurrence [16]. Implementation of the DLNM modeling framework [7] is available in the statistical environment R [17]. Based on the methodology presented in Gasparrini et al. [9], we modelled the exposure-response curve using a quadratic B-spline with three internal knots placed at the 10th, 75th, and 90th percentiles of the observed temperature distribution, and the lag-response curve (with maximum lag up to 21 days) using a natural cubic B-spline with an intercept and three internal knots equally spaced on the log scale. In order to control for seasonal and long-term effects, we included a natural cubic B-spline of time with 8° of freedom per year. Finally, the model also included a categorical variable indicating the day of the week.

The quasi-Poisson regression model with no pollutants included is specified as follows:

$$ E\left({Y}_t\right)= \exp \left\{\alpha +\beta {T}_{t,l}+NS\left( time,\ df\right)+\lambda DO{W}_t\right\} $$

where *E(Y*_*t*_*)* is expected daily death count on day *t*; *α* is the intercept; *T*_*t,l*_ is a matrix of variables obtained by the transformation of mean daily temperature, *β* is a vector of coefficients for *T*_*t,l*_, and *l* is the lag day; *NS(time,df)* is the natural cubic spline of time; and *DOW*_*t*_ and *λ* are dummy variables representing the day of the week and the corresponding vector of coefficients, respectively. Quasi-Poisson was used to compensate for overdispersion since the residual deviance was larger than the residual degrees of freedom (the dispersion parameter was 1.06). We shall refer to this model in further text as the base model.

## Results and discussion

**Fig. 1:**
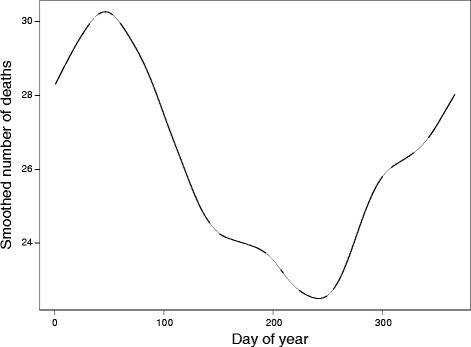
Smoothed empirical mortality annual variations for the period 2009–2014. Mortality variations exhibit seasonal pattern, with peak values in the middle of February and minimum values around the start of September

The analysis included a total of 56,920 residents aged 65 years or older who died from circulatory (93.27 %) and respiratory diseases (6.73 %). As shown in Fig. 1, mortality annual variations for the entire period exhibited a strong seasonal pattern, with peak values in the middle of February and minimum values around September, 1st. Two minor increases occurred at the beginning of July and the end of October, suggesting the potential impact of sudden temperature changes on human health. The figures presenting circulatory and respiratory daily mortality segmented by age and gender, and their seasonal variations are shown in Additional files 1 and 2.

The climate in Belgrade is moderate continental with all four seasons. During the study period, mean daily temperatures were in the range from −17.4 to 30.1 °C, with the average value of 11.9 °C. The average number of days with temperatures below 0 °C was 40 per year, whereas the coldest and warmest months were January and July, with 0.4 and 22.2 °C, respectively. The average seasonal temperatures for the entire period were 12.1, 21.4, 12.7 and 1.1 °C for the spring, summer, autumn and winter, respectively.

The daily mean concentrations of PM_10_, NO_2_, SO_2_ and soot for the entire period were 48.3, 32.7, 15.5 and 20.7 μg m^−3^, respectively. The number of days with average PM_10_ concentrations exceeding 50 μg m^−3^ was in the range from 75 to 155 per year, which is considerably higher than the Air Quality Standard margin (35 exceedances per year), whereas the mean annual NO_2_ concentrations did not exceed the value of 40 μg m^−3^, nor any mean daily SO_2_ levels higher than the recommended limit of 135 μg m^−3^ were observed.

The levels of PM_10_ and SO_2_ exhibited a clear seasonal pattern, with highest concentrations observed during cold season (Additional file 3), whereas the seasonality of soot was less pronounced. Furthermore, the moving average PM_10_ and SO_2_ concentrations were highly correlated, suggesting that the variations of two pollutants, particularly those long-term, were driven by the same anthropogenic and meteorological factors. Conversely, NO_2_ concentrations exhibited pronounced weekly, but not seasonal variations, which can be partly attributed to the traffic-related emissions that are present throughout the year.

**Fig. 2:**
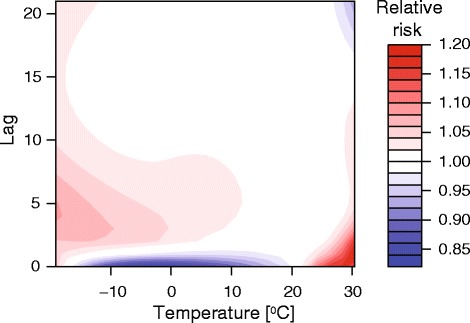
Plot of the exposure-lag-response relationship between temperature and all-cause mortality, with reference at 21 °C. The effects of heat are observed on the same day or after 1–3 days, whereas the effects of cold were distributed across few days of exposure

The lag-specific effects of temperature on mortality obtained from the base model are provided in Fig. 2. As can be seen, the heat-related mortality appears to be an acute event followed by a reduction in death rates, with maximum effects observed on the same day or lags of 1–3 days. Nevertheless, the effects of cold spells were observed to be more evenly distributed across 3–6 days of exposure with less evidence of subsequent harvesting, which complies with previous studies reporting sustained health effects of low temperatures [18].

**Fig. 3:**
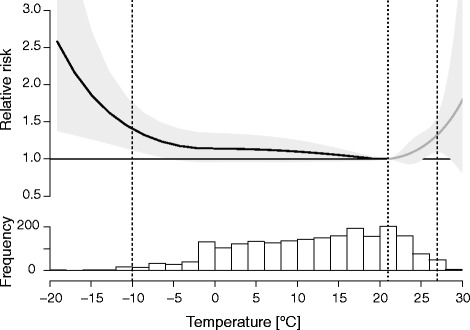
Overall cumulative exposure-response curve and temperature distribution in Belgrade 2009–2014. Dotted and dashed lines represent optimum and extreme temperatures, respectively

As regards the cumulative effects of temperature over the entire lag period, the U-shaped exposure-response curve was identified as the most representative. The minimum relative risk corresponding to the optimum temperature of 21 °C is presented in Fig. 3 as a dotted line, whereas the cut-off values at the 1st and 99th percentile of the temperature distribution, which stand for extreme cold and heat, are displayed as dashed vertical lines.

According to the findings of Gasparrini et al. [9], cold temperatures were responsible for advancing a substantial fraction of deaths (7.29 %), compared to hot days (0.42 %), whereas most deaths were attributable to exposure to moderate temperatures. As shown in Fig. 3, the highest relative risk was observed for a small proportion of extremely cold days, whereas the estimated effect of the days with moderate temperatures, corresponding to the smooth response, was also noticeable.

Upon estimating the effects of temperature on mortality, we proceeded to examine the effects of air pollutants. The strong positive correlations between PM_10_, SO_2_ and soot (Table 1) suggest that these species share the same origin, and thus, the effects of some pollutants may distort the observable patterns of others.

**Table 1:** Pollutant correlations for the entire period, and for cold and warm season

	PM_10_	NO_2_	SO_2_	Soot	PM_10_	NO_2_	SO_2_	Soot
PM_10_	1				1	0.34	0.43	0.27
NO_2_	0.50	1			0.64	1	0.40	0.20
SO_2_	0.67	0.36	1		0.65	0.44	1	0.23
Soot	0.66	0.44	0.56	1	0.74	0.58	0.54	1

Pollutant correlations for the entire period (left), and for cold and warm season (right, lower and upper triangular, respectively)

Given that the extreme cold events and increased levels of pollutants often coincide, it is necessary to understand the combined effects of these two factors on mortality. We have developed our modeling strategy relying on the following logical sequence:

1. Increased concentrations of air pollutants (at least within their normal range of concentrations) require a certain amount of time to reach their cumulative effect on mortality, the time being not less than 24 h, and thus, daily average is the level of aggregation of the measurement data used in this and numerous other studies. This cumulative effect is expected to be more stable, and thus, statistically robust in comparison to short-term lag-specific effects (referring to lags of up to 7 days).
2. Up to the n^th^ period (day), the longer the high values of pollutants persist, the greater the cumulative effect on mortality. As the cumulative effect on mortality reaches its peak (period n), it largely depletes the pool of those susceptible in the observed population, as their death is being brought forward by a matter of a few days.
3. Owing to the aforementioned harvesting effect, the cumulative primary effect of the pollutant starts to decrease afterward, reaching its minimum value on day n1.

Based on these suppositions, two variables were used to describe the temporal distribution of the cumulative effect of each pollutant. The first variable represented the average pollutant concentrations during the specific timeframe at the end of which (day n) the primary effect of the pollutant on mortality reaches its local maximum. The second variable is defined to capture the temporal positioning of the minimum rate of mortality (day n1) attributable to the post-harvesting reduction in death rates. Accordingly, the cumulative effects of each pollutant are included as two simple moving averages (*SMA*) of its observed concentrations in 10 μg m^−3^ (*P*) over *n* consecutive days, ending on-and inclusive of-day *t*.

$$ SMA{(P)}_{t,n}=\frac{1}{n}{\displaystyle \sum_{i=1}^n}{P}_{\left(t-i\right)+1} $$

Rather than assume any particular temporal distribution of the effect, as is typically done in the literature, we visually inspected the values of regression coefficient estimates that relate SMA concentrations of each of the four air pollutants to mortality rates within a reasonable timeframe of 60 days, seeking for “peak and trough” temporal patterns that can be attributed to the corresponding harvesting effects of the specific pollutant. The temporal pattern was expected to look as follows: the relative risk should start off at a value close to 1 and stay relatively close to 1 for short time periods (no major effect is expected within the first few days), then steadily increase to a finite value within a reasonable timeframe, and lastly, display a steady decline to a value below 1, with the decline roughly matching the timing and magnitude of the preceding increase. The duration period of the effect depends on the specific system characteristics, such as population susceptibility, pollutant concentrations and their spatio-temporal dynamics.

**Fig. 4:**
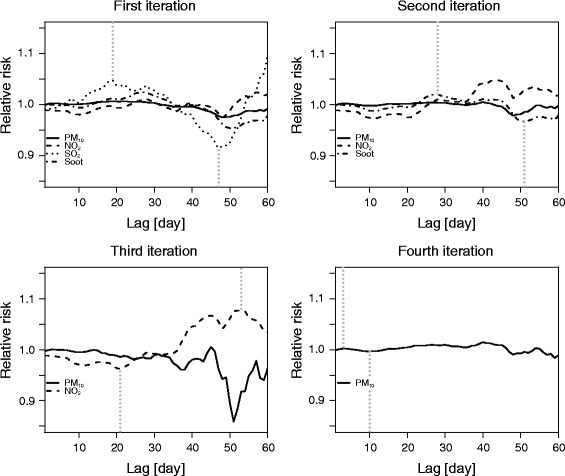
The procedure for including air pollutants in the final model. The estimation of the timeframe-specific relative risk was repeated each time after exclusion of the pollutant with the most clear harvesting effect pattern. Dotted lines refer to timeframes that are associated with highest positive or negative relative risk

As certain pattern deviations were expected due to strong correlations (referring to SMA values and not daily observations), we included the pollutants in sequence iteratively, giving priority to those with stronger effects (as estimated by the assessed relative risk) and clearer temporal patterns of these effects. Figure 4 shows the procedure for determining the order of pollutant inclusion in the final model. Lags 0–60 refer to timeframes for which the relative risk was estimated by including one by one timeframe-specific SMA pollutant concentration in the model. As can be seen, once the clear harvesting effect pattern had been identified and attributed to a specific pollutant, the pollutant was added to the final model, and the estimation of the relative risk was repeated for the remaining pollutants.

As can be seen, the effects of pollutants were relatively stable between iterations due to the fact that SMA were used instead of lag values. The final model is specified as follows:

$$ \begin{array}{l}E\left({Y}_t\right)= \exp \left\{\alpha +\beta {T}_{t,l}+NS\left( time,\ df\right)+\lambda DO{W}_t+{\gamma}_1SMA{\left({\mathrm{SO}}_2\right)}_{t,19}+{\gamma}_2SMA{\left({\mathrm{SO}}_2\right)}_{t,47}\right.\hfill \\ {}\kern10.5em +{\gamma}_3SMA{\left(\mathrm{Soot}\right)}_{t,28}+{\gamma}_4SMA{\left(\mathrm{Soot}\right)}_{t,51}+{\gamma}_5SMA{\left({\mathrm{NO}}_2\right)}_{t,53}+{\gamma}_6SMA{\left({\mathrm{NO}}_2\right)}_{t,21}\hfill \\ {}\left.\kern10.5em +{\gamma}_7SMA{\left({\mathrm{PM}}_{10}\right)}_{t,3}+{\gamma}_8SMA{\left({\mathrm{PM}}_{10}\right)}_{t,10}\right\}\hfill \end{array} $$

The detailed model estimates are presented in Additional file 4, in the columns labeled “Saturated model”.

The risk of excess death with short-term exposure to elevated concentrations of PM_10_ was observed, whereas for SO_2_ and soot, the marked effects were found within 2–3 weeks of exposure. A 10 μg m^−3^ increase in PM_10_ concentrations is followed by a negligible increase in mortality (0.04 %), whereas a moderate increase in death rates in the range from 0.7 to 1.3 % was associated with the same increment in NO_2_, SO_2_ and soot concentrations. Comparable short-term health effects of exposure to PM were also reported in the extensive review of Brook et al. [19]. They concluded that the elevated PM_2.5_ concentrations over 5 days lead to the increase in circulatory mortality risk by 0.4 % to 1.0 %. Thereby, it is important to mention that the health effects of PM_2.5_ are more severe compared to PM_10_, because the particles of smaller diameter penetrate deeper in the respiratory tract. The air pollution-related mortality estimates also comply with the findings of other extensive studies [20, 21], reporting moderate increases in mortality associated with the exposure to PM, SO_2_ and NO_2_. As can be seen in Fig. 4, the effect pattern was inverted in the case of NO_2_, which could indicate that the exposure to high NO_2_ levels reaches their maximum effect (day n) beyond the examined time period. This complies with the results of our previous study [13], aimed at investigating the association between short- and medium-term (up to 90 days) exposure to nitrogen dioxide (NO_2_) and mortality within the several timeframes. As shown, the short-term exposure to NO_2_ exhibited negative associations with death rates, whereas the medium-term exposure to NO_2_ was associated with significant increase in mortality.

Furthermore, it could be assumed that the effects attributed to NO_2_ exposure are not realistic, but rather a result of omitted variable bias, which is even more likely taking into account its considerable relative importance (Fig. 6), which was highest of all species. Likewise, Brook et al. [22] reported surprisingly strong relationship between NO_2_ and non-accidental mortality across 10 cities in Canada. They assumed that NO_2_ concentrations were acting as an indicator of some other variable or process, because NO_2_ levels exhibit high correlations in time and space with a range of toxic traffic-related species, including volatile organic compounds and polycyclic aromatic hydrocarbons, which could also contribute to the observed effects.

**Fig. 5:**
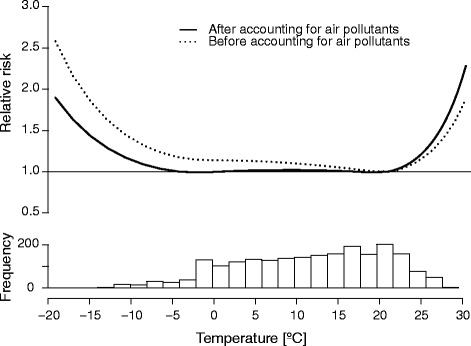
Overall cumulative exposure-response curve after accounting for the effects of air pollution and temperature distribution. After adjusting for air pollution, the optimum temperature range was relatively broad

In Fig. 5, the overall effects of temperature on mortality before and after accounting for air pollutant concentrations are compared.

After accounting for the effects of air pollutants, estimates of backward attributable risk of non-optimal temperature decreased from 8.80 % (5,009 people) to 3.00 % (1,706 people) (for detailed information on calculation of attributable risk see [23]). More specifically, the base model results showed that the exposure to cold temperatures was associated with significantly higher risk (7.35 %), compared to hot days (1.50 %). However, after adjusting for air pollutants, the relative risk associated with cold temperatures was lowered to 1.06 %, whereas the risk of heat-related mortality slightly increased to 1.96 %. When adopting a backward perspective, the risks are simultaneously computed for the same time *t*, and thus, negligible difference ($b-A{F}_{x,t}^{r_{1+}{r}_2}\le\ b-{A}_{x,t}^{r_1} + b-{A}_{x,t}^{r_2}$) could be observed between the sum of components (1.06 % and 1.96 %) and the overall attributable risk (3.00 %) [23]. It is also worth noting that after including air pollutants in the model, the optimum temperature range was relatively broad, from −5 to 21 °C, which differs from previous findings that most of related deaths were caused by moderate temperatures [9]. Correspondingly, the relative contributions of extreme temperature events have also changed (Table 2).

**Table 2:** Relative risk for the 1 and 99th percentile of the observed temperature distribution (95 % CI)

Temperature	Before accounting for the effects of air pollutants	After accounting for the effects of air pollutants
−10.0 °C	1.40 (1.12, 1.76)	1.14 (0.86, 1.51)
27.0 °C	1.32 (1.10, 1.60)	1.45 (1.20, 1.76)

Anderson and Bell [24] showed that the temperature-related mortality risk was slightly lowered (approx. 0.4 % on average) after controlling for the effects of O_3_ and PM_10_. As shown in Additional file 5, the change in mortality risk after accounting for the short-term lag-effects of air pollutants was negligible. However, after accounting for the cumulative effects of air pollutants, as proposed herein, the significant change in estimated risk was observed.

As part of a supplementary analysis, and with the aim of arriving at a more parsimonious model specification, we performed optimization with respect to information criteria, and investigated whether a model with a double-threshold parameterization can be used as a reasonable approximation to the saturated model. Firstly, we examined whether some of the model terms could be left out without substantial loss to the model fit. In order to gauge the relative quality of the model in terms of the goodness-of-fit/complexity trade-off, we used a quasi-likelihood (we used the C-hat [the dispersion parameter] value obtained from the saturated model, which is 1.06) counterpart of Akaike’s information criterion (QAICc), specifically its implementation available in the GLMULTI package in R [25]. An exhaustive screening, which included each of the 2,048 (2^11^) possible models for the specified set of variables, showed that the best model, in terms of the information criteria, is the following:

$$ \begin{array}{l}E\left({Y}_t\right)= \exp \left\{\alpha +\beta {T}_{t,l}+NS\left( time,\ df\right)+{\gamma}_1SMA{\left({\mathrm{SO}}_2\right)}_{t,19}+{\gamma}_2SMA{\left({\mathrm{SO}}_2\right)}_{t,47}+{\gamma}_3SMA{\left(\mathrm{Soot}\right)}_{t,28}\right.\hfill \\ {}\left.\kern10.5em +{\gamma}_4SMA{\left(\mathrm{Soot}\right)}_{t,51}+{\gamma}_5SMA{\left({\mathrm{NO}}_2\right)}_{t,53}+{\gamma}_6SMA{\left({\mathrm{NO}}_2\right)}_{t,21}\right\}\hfill \end{array} $$

Detailed model estimates are presented in Additional file 4, “Optimal model” columns.

**Fig. 6:**
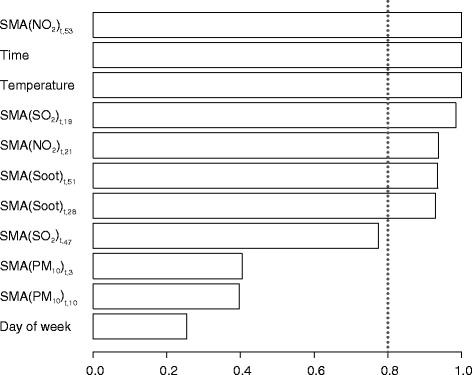
Model-averaged relevance of terms. Dotted line represents threshold importance

The screening process also enabled an insight into the estimated importance of each of the variables. The estimated importance was calculated as the sum of the relative evidence weights of all models in which the term appears. Relative evidence weights are computed as exp(ΔIC/2), where ΔIC is the difference in IC between a model and the best model, and they are normalized so that they sum up to one. They can be interpreted as probabilities for each model to be the best in the set. Given the fact that PM_10_ concentrations have the smallest estimated relative importance of all examined species (Fig. 6), including only PM_10_ in the form of a 0–1 lag variable, as performed in research [26] is clearly not the most effective way to control for the effects of air pollution.

Since the saturated model indicates the existence of an optimum temperature range, it is valuable to know whether a model with the double-threshold parameterization (with −5 to 21 °C being the thresholds) could be used as a simple approximation to it, without significant loss to the model fit. The analysis showed, however, that the loss in the model fit, from 0.2832 for saturated to 0.2574 for double-threshold model as measured by correlation between the predicted and the observed values (pseudo R squared), was not compensated for in terms of information criteria—QAICc increased from 12518.25 to 12553.38. Visual inspection indicated that the linear components of the double-threshold model are incapable of accurately describing the progressive nature of the effects of extreme temperatures.

In comparison to the herein proposed SMA method, DLNM framework is technically more sophisticated and more appropriate for the exploration of the particular lag-specific and exposure-level-specific pollutant effects. It is also superior when long-time series are available. However, given that DLNM framework does not employ the concept of a stable cumulative effects between pollution and mortality, but rather tries to describe the entire exposure-lag-response association, it is demanding regarding the number of parameters that need to be estimated. For instance, using DLNM for describing the effect of temperature on mortality would involve estimating a total of 5 × 5 = 25 parameters.^1^ Furthermore, describing the effects of four air pollutants would require an additional 100 parameters on top of the 80 parameters already included in the model (25 parameters are estimated for describing the effect of temperature, 8 × 6 years = 48 parameters for capturing seasonal and long-term trend, 6 parameters for day of the week, and 1 overall intercept). Conversely, the herein proposed procedure requires 8 + 8 = 16 parameters.

Furthermore, the herein proposed SMA method is the most parsimonious method that is consistent with the assumption that significant cumulative, medium-term effects of air pollutant levels on mortality exist. If this assumption is valid, which has been confirmed by previous studies, then it suffices to include only two properly selected SMA terms for each pollutant that captures the majority of their respective effects. The choice of SMA lags describes the temporal positioning of the cumulative effect’s peak, while the regression estimates of the two corresponding regression coefficients describe the magnitude of the effect for each particular air pollutant. Hence, in the case of short-time series, when air pollutants are included into the research model solely to account for their effect on mortality, the SMA approach may be comparatively more efficient than DLNM.

The results of using the DLNM to model the effects of four pollutants are presented in Additional file 6. As can be seen, the observed shapes of the effects confirm the central hypothesis of our study, which is that, besides the well-evidenced short-term effects of air pollution on mortality (most visible in the case of soot), significant cumulative (perhaps even seasonal) medium-term effects of air pollutant levels on mortality (most visibly in the case of SO_2_) are observed. The harvesting effect pattern is also clearly visible in both short- and medium-term effects. In Additional file 7, temperature-related mortality risk is presented, after accounting for air pollutants using SMA and DLNM.

Some general limitations of retrospective epidemiological studies aimed at health risk estimation are also applicable to this study. By analyzing the effects of air pollution and temperature, the results obtained from a number of central monitoring stations are assumed to be representative of individual exposure, although people have different activity patterns and spend a significant period of time indoors. Furthermore, certain species are selected as indicators of air pollution, although pollution refers to a complex mixture of gaseous species and particles that permanently interact and undergo physico-chemical transformations [27]. However, regardless of the potential limitations, the results of retrospective epidemiological studies based on large-sample statistics remain an important source of information for designing environmental regulations [28]. The advantage of the herein proposed method is that it includes the cumulative mid-term effects of air pollution in the traditional Poisson regression model in a parsimonious way, which complies with the nature of their impact. The limitation of the proposed method is possible overfitting due to the limited number of observations, which needs to be eliminated through further studies.

## Conclusions

The herein proposed method is based on the theoretical premise that there are significant cumulative mid-term effects of air pollution on mortality, which are more stable, and thus more statistically robust, than the lag-specific effects commonly included in regression models. The proposed method requires the researcher to visually identify a “peak and trough” temporal pattern for the effects of each of the pollutants, which is then included in the model using only two parameters, yielding a rather parsimonious model specification.

The presented results suggest that the inference that cold-attributable mortality currently accounts for one order of magnitude more deaths than mortality associated with heatwaves might not be globally applicable. Accounted for in an appropriate way, the effects of air pollutants explained most of the mortality previously associated with cold temperatures, and a greater temperature-attributable mortality burden was associated with the contribution of heat.

The more realistic estimates of the mortality risk associated with extreme temperature events becomes increasingly important for planning of future public health interventions and adaptation measures, with the global warming impact that is already underway. However, further research is needed to identify all environmental risk factors and clarify their complex associations, which have been largely obscured by the current approach.

## Abbreviations

PM_10_, particles smaller than 10 μm; NO_2_, nitrogen dioxide; SO_2_, sulfur dioxide; CI, confidence interval

## Additional files

Additional file 1: Circulatory and respiratory daily death rates segmented by age and gender. (DOCX 71 kb) Additional file 2: Seasonal variations in circulatory and respiratory daily death rates. (DOCX 154 kb) Additional file 3: Seasonal variations in pollutant concentrations. (DOCX 129 kb) Additional file 4: Model estimates. (DOCX 15 kb) Additional file 5: Temperature-related mortality risk estimates after accounting for the short-term lag-effects of air pollutants and cumulative effects of air pollutants (as proposed herein). (DOCX 158 kb) Additional file 6: The effects of four pollutants modeled using DLNM framework. (DOCX 662 kb) Additional file 7: Temperature-related mortality risk, after accounting for air pollutants using SMA and DLNM. (DOCX 188 kb)

## Footnotes

1 We used a quadratic B-spline with three internal knots and no intercept for the exposure-response curve: 3 df for knots + 2 df for the degree of the piecewise polynomial (quadratic) + 0 df for intercept (no intercept) = 5 df, and a natural cubic B-spline with three internal knots and an intercept for the lag-response curve: 3 df for knots + 1 df for intercept + 1 df = 5 df.
