# Supplementary figures and images for: Temperature-related mortality estimates after accounting for the cumulative effects of air pollution in an urban area
Source: Environ Health. 2016 Jul 11;15:73. doi: 10.1186/s12940-016-0164-6 (PMC4940758; doi:10.1186/s12940-016-0164-6)

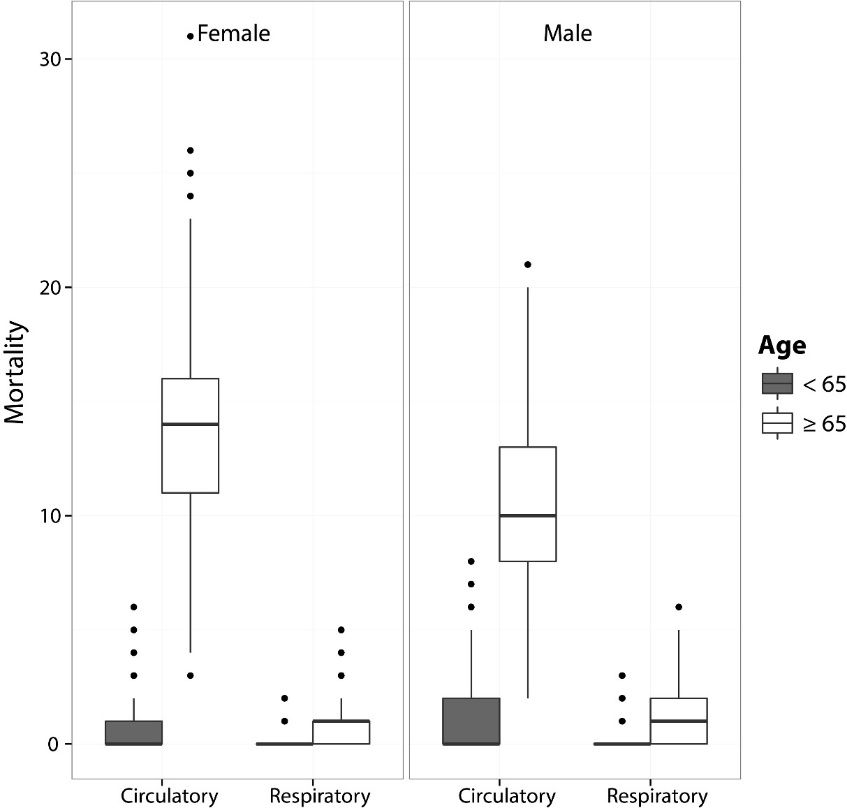


**Figure A1** Circulatory and respiratory daily death rates segmented by age and gender

Supplement: Additional file 1: — Circulatory and respiratory daily death rates segmented by age and gender. (DOCX 71 kb) [file 12940_2016_164_MOESM1_ESM.docx]

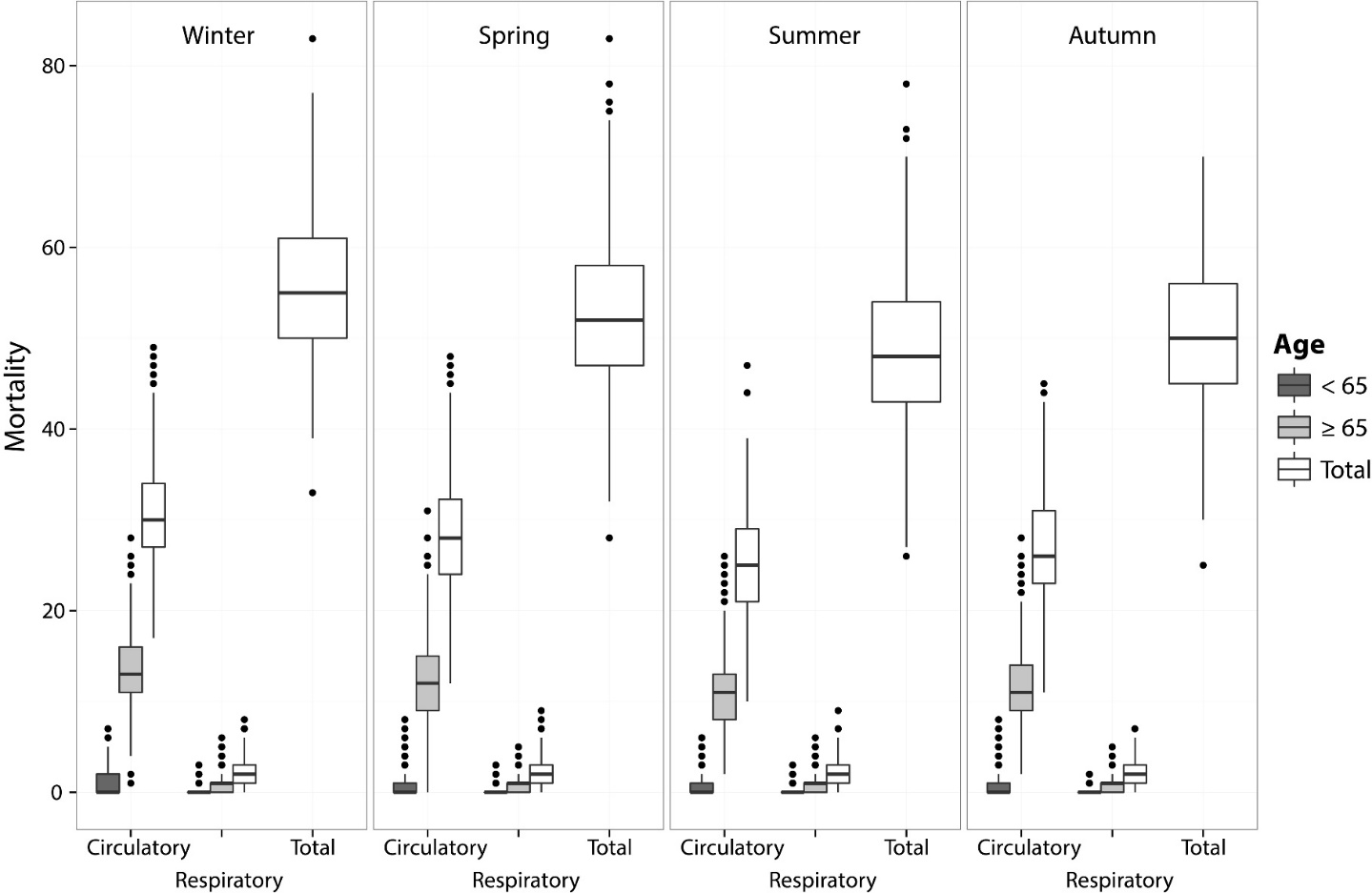


**Figure A2** Seasonal variations in circulatory and respiratory daily death rates

Supplement: Additional file 2: — Seasonal variations in circulatory and respiratory daily death rates. (DOCX 154 kb) [file 12940_2016_164_MOESM2_ESM.docx]

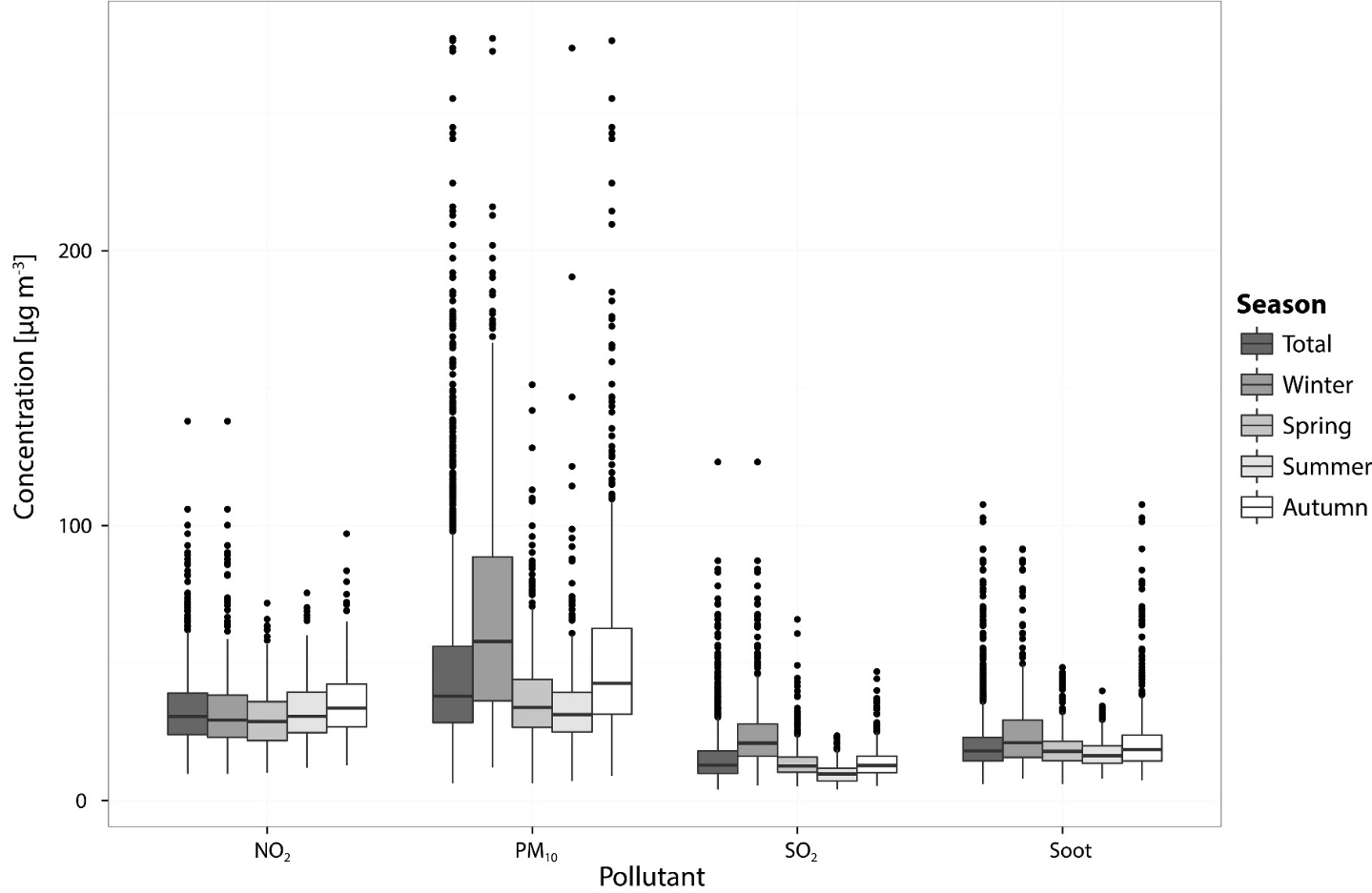


**Figure A3** Seasonal variations in pollutant concentrations

Supplement: Additional file 3: — Seasonal variations in pollutant concentrations. (DOCX 129 kb) [file 12940_2016_164_MOESM3_ESM.docx]

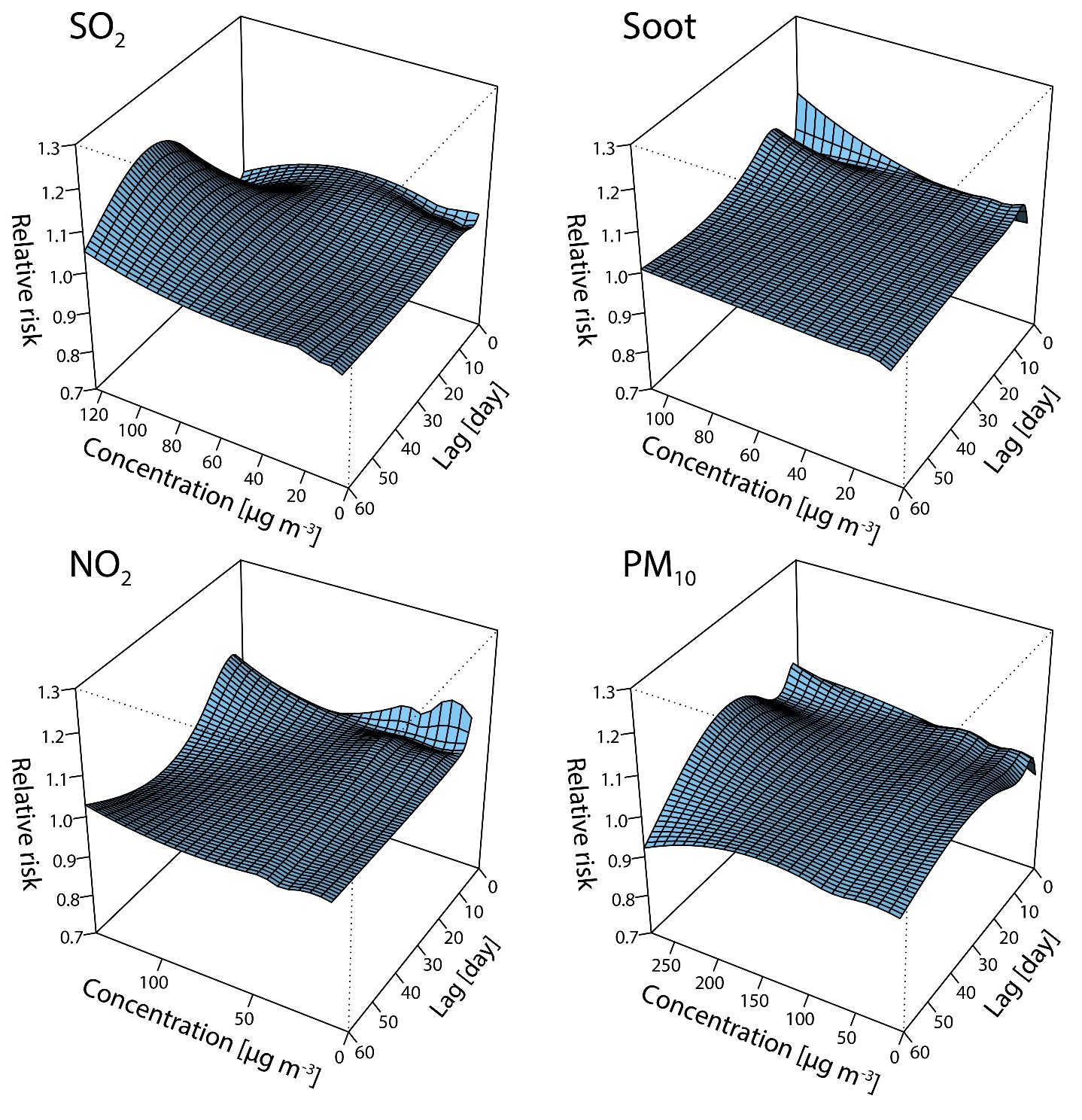


**Figure A5** The effects of four pollutants modeled using DLNM framework

Supplement: Additional file 6: — The effects of four pollutants modeled using DLNM framework. (DOCX 662 kb) [file 12940_2016_164_MOESM6_ESM.docx]
