# Supplementary material for: Temperature-related mortality estimates after accounting for the cumulative effects of air pollution in an urban area
Source: Environ Health. 2016 Jul 11;15:73. doi: 10.1186/s12940-016-0164-6 (PMC4940758; doi:10.1186/s12940-016-0164-6)
Supplement: Additional file 4: — Model estimates. (DOCX 15 kb) [file 12940_2016_164_MOESM4_ESM.docx]

**ADDITIONAL FILE**

**Table A1** Model estimates

|  | **MODEL ESTIMATES** | | **EXPONENTIATED VALUES (RR)** | |
| --- | --- | --- | --- | --- |
|  | Saturated model | Optimal model (in terms of QAICc) | Saturated model | Optimal model (in terms of QAICc) |
| Tuesday | -0.023 (-0.055, 0.009) |  | 0.9773 (0.9465, 1.009) |  |
| Wednesday | -0.027* (-0.059, 0.005) |  | 0.9734 (0.9427, 1.005) |  |
| Thursday | -0.022 (-0.054, 0.010) |  | 0.9782 (0.9474, 1.0101) |  |
| Friday | -0.013 (-0.045, 0.019) |  | 0.9871 (0.956, 1.0192) |  |
| Saturday | -0.028* (-0.061, 0.004) |  | 0.9724 (0.9408, 1.004) |  |
| Sunday | 0.013 (-0.019, 0.044) |  | 1.0131 (0.9812, 1.045) |  |
| SMA(SO_2_)_t,19_ | 0.013*** (0.006, 0.020) | 0.012*** (0.006, 0.019) | 1.0131 (1.006, 1.0202) | 1.0121 (1.006, 1.0192) |
| SMA(SO_2_)_t,47_ | -0.013** (-0.026, -0.001) | -0.014** (-0.026, -0.001) | 0.9871 (0.9743, 0.999) | 0.9861 (0.9743, 0.999) |
| SMA(Soot)_t,28_ | 0.007*** (0.003, 0.012) | 0.007*** (0.002, 0.011) | 1.0070 (1.003, 1.0121) | 1.0070 (1.002, 1.0111) |
| SMA(Soot)_t,51_ | -0.014*** (-0.024, -0.005) | -0.014*** (-0.024, -0.005) | 0.9861 (0.9763, 0.995) | 0.9861 (0.9763, 0.995) |
| SMA(NO_2_)_t,53_ | 0.013*** (0.004, 0.023) | 0.014*** (0.004, 0.023) | 1.0131 (1.004, 1.0233) | 1.0141 (1.004, 1.0233) |
| SMA(NO_2_)_t,21_ | -0.006*** (-0.011, -0.002) | -0.007*** (-0.011, -0.002) | 0.9940 (0.9891, 0.998) | 0.9930 (0.9891, 0.998) |
| SMA(PM_10_)_t,3_ | 0.0004 (-0.0001, 0.001) |  | 1.0004 (0.9999, 1.001) |  |
| SMA(PM_10_)_t,10_ | -0.001 (-0.002, 0.0002) |  | 0.9990 (0.998, 1.0002) |  |
| Constant | 5.184*** (0.899, 9.469) | 5.382*** (1.093, 9.671) | 178.3950 (2.4571, 12951.9288) | 217.4568 (2.9832, 15851.1923) |
| Observations | 2,136 | 2,136 | 2,136 | 2,136 |

Notes: ***p < 0.01; **p < 0.05; *p <0 .1
