# Supplementary material for: Temperature-related mortality estimates after accounting for the cumulative effects of air pollution in an urban area
Source: Environ Health. 2016 Jul 11;15:73. doi: 10.1186/s12940-016-0164-6 (PMC4940758; doi:10.1186/s12940-016-0164-6)
Supplement: Additional file 5: — Temperature-related mortality risk estimates after accounting for the short-term lag-effects of air pollutants and cumulative effects of air pollutants (as proposed herein). (DOCX 158 kb) [file 12940_2016_164_MOESM5_ESM.docx]

**
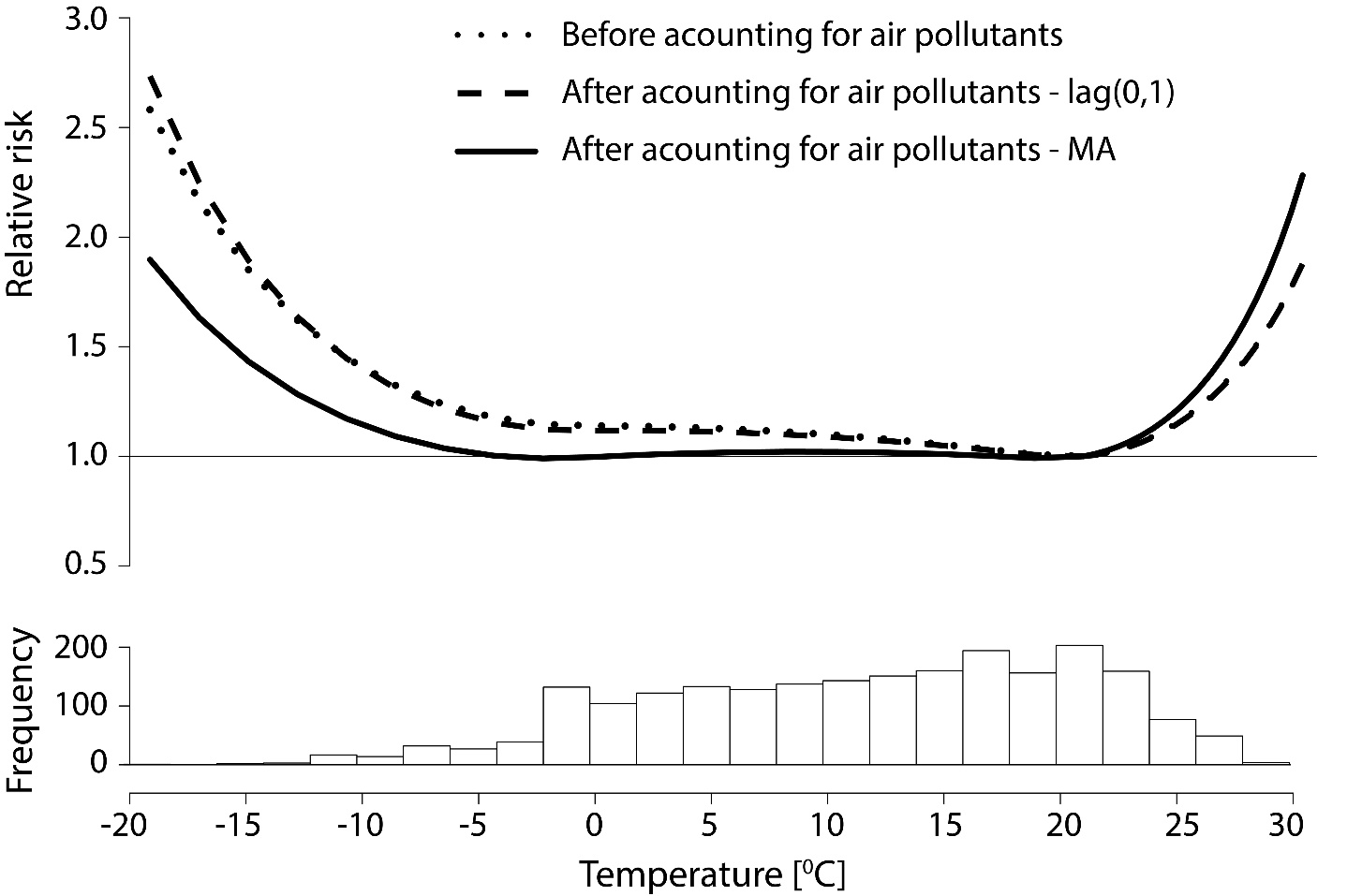
**

**Figure A4** Temperature-related mortality risk estimates after accounting for the short-term lag-effects of air pollutants and cumulative effects of air pollutants (as proposed herein)
