# Supplementary material for: Temperature-related mortality estimates after accounting for the cumulative effects of air pollution in an urban area
Source: Environ Health. 2016 Jul 11;15:73. doi: 10.1186/s12940-016-0164-6 (PMC4940758; doi:10.1186/s12940-016-0164-6)
Supplement: Additional file 7: — Temperature-related mortality risk, after accounting for air pollutants using SMA and DLNM. (DOCX 188 kb) [file 12940_2016_164_MOESM7_ESM.docx]

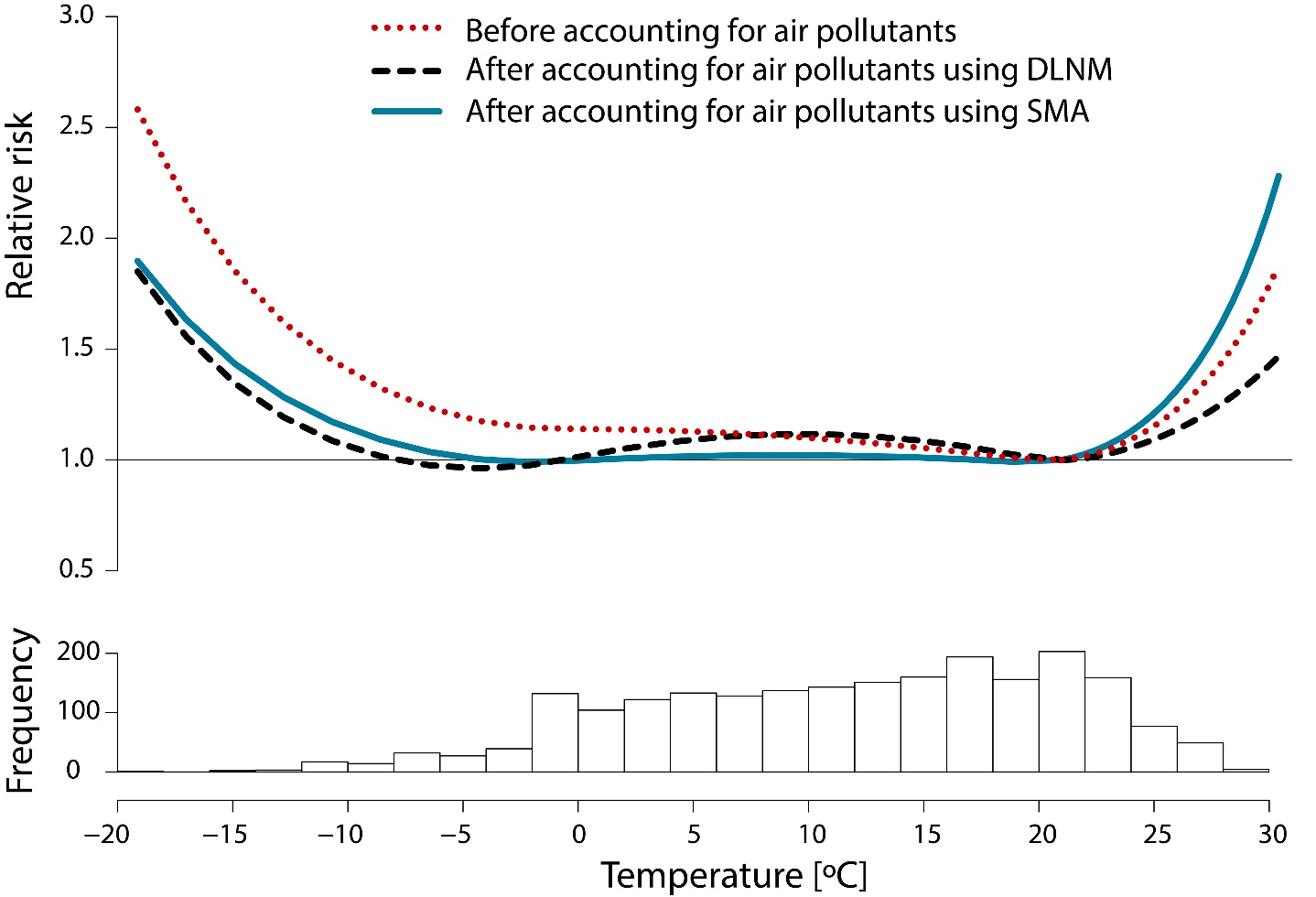


**Figure A6** Temperature-related mortality risk, after accounting for air pollutants using SMA and DLNM
